# Comparison of microwave ablation versus lauromacrogol injection ablation for 50–75% cystic thyroid nodules: a two-center retrospective study

**DOI:** 10.3389/fendo.2026.1751988

**Published:** 2026-03-10

**Authors:** Jiayan Bao, Wenbo Ding, Zheng Zhang, Yanwei Chen, Shuangshuang Zhao, Yun Cai, Wenjun Li, Jiwen Qian, Feng Zhao, Baoding Chen

**Affiliations:** 1 Department of Medical Ultrasound, Cancer Institute of Jiangsu University, Affiliated Hospital of Jiangsu University, Zhenjiang, China; 2 Department of Ultrasound, the Affiliated Hospital of Integrated Traditional Chinese and Western Medicine, Nanjing University of Chinese Medicine, Nanjing, China

**Keywords:** ablation, microwave, lauromacrogol, predominantly cystic thyroid nodules, cystic components, ultrasound

## Abstract

**Purpose:**

To compare the efficacy between microwave ablation (MWA) and lauromacrogol injection ablation (LIA) for treating 50-75% cystic thyroid nodules and systematically identify the factors influencing outcomes.

**Materials and methods:**

This retrospective study included 106 patients with predominantly cystic thyroid nodules (PCTNs) with 50-75% proportion of cystic components (simple as 50–75% cystic thyroid nodules) who underwent ultrasound-guided MWA (n=51) or LIA (n=55) in two hospitals between April 2017 and November 2023. The primary endpoint was 12-month volume reduction rate (VRR). ANCOVA was used to compare adjusted 12-month VRR between groups after adjusting for confounders. Secondary endpoints included effective treatment rate (VRR >50% at 6 or 12 months), recurrence, and stratified analysis analyses by initial nodule volume (>10 ml and ≤10 ml) and vascularity (Grades 0–1 and 2-3). Regrowth-free survival was estimated by Kaplan-Meier (KM) analysis and compared with the Log-rank test.

**Results:**

At 12 months postoperatively, the mean VRR for MWA was 91.5 ± 9.8% and 81.1 ± 2.4% for LIA (F = 4.40, P = 0.005). MWA yielded higher VRR than LIA at 6 and 12 months across both low- and high-vascularity subgroups (P<0.05). For nodules >10 ml, MWA produced significantly greater VRR at 3, 6 and 12 months (P<0.05); no significant difference was observed for nodules ≤10 ml (P>0.05). The 12-month effective treatment rate was 96.1% (49/51) after MWA versus 81.8% (46/55) after LIA (P = 0.018). Regrowth rates were 3.9% (2/51) for MWA and 20.0% (11/55) for LIA (P = 0.006). The KM analysis showed significantly longer regrowth-free survival following MWA (Log-rank P = 0.010).

**Conclusion:**

Both MWA and LIA exhibit favorable efficacy in the treatment of 50–75% cystic thyroid nodules. Importantly, based on the approximately 12-month follow-up, MWA demonstrates superior efficacy and improved control of regrowth compared to LIA. This advantage of MWA is further amplified in the management of larger nodules.

## 1 Introduction

The widespread application of high-resolution ultrasound has markedly increased the detection of thyroid nodules (TNs), with prevalence estimates now ranging from 25% to 70% (1).

Benign thyroid nodules (BTNs) represent approximately 90% of clinically detected TNs; among these, cystic and predominantly cystic thyroid nodules (PCTNs) account for 15-30% (2, 3).

PCTNs of small size are often asymptomatic, but larger nodules (diameter >3–4 cm) frequently cause compressive symptoms such as dyspnea or dysphagia. In some cases, capsular vessel rupture and acute intranodular hemorrhage can precipitate rapid nodule enlargement, painful neck swelling, or cosmetic concerns (1, 4, 5).

The 2015 American Thyroid Association (ATA) guideline recommend routine clinical monitoring of PCTNs, but intervention is required in the presence of compression, suspicious cytopathology, or patient anxiety (3). It is worth noting that some studies have shown that although most of PCTNs behave as benign lesions, molecular alterations such as BRAF and TERT promoter mutations and dysregulation angiogenic signaling, which are associated with aggressive thyroid cancer, have been recognized as important markers for risk stratification (6).Owing to the invasive nature of conventional surgical interventions, which carry risks including recurrent laryngeal nerve injury, parathyroid dysfunction, hypothyroidism, and scarring, ultrasound-guided ablation therapy has become the clinical recommendation for PCTNs as a minimally invasive alternative offering safety without compromising efficacy (7–9).

Ultrasound-guided percutaneous ethanol ablation (EA) is the most frequently used chemical ablation method for treating cystic thyroid nodules and PCTNs (10–12). However, it has been reported that lauromacrogol injection for ablation (LIA) demonstrates a superior safety profile compared to traditional EA in the management of PCTNs, with comparable efficacy, and significantly reduces postoperative pain (58-72% reduction among studies) and complications (13–16). Consequently, LIA demonstrates potential as a viable alternative to EA in the management of benign cystic thyroid nodules and PCTNs (17–19). Nevertheless, in the case of dense solid portions, it is difficult for the sclerosing agent to penetrate adequately, resulting in higher recurrence rates. For these nodules, the 2020 European Thyroid Association guideline recommends thermal ablation for those who fail to undergo secondary ablation (20). As a thermal technique, microwave ablation (MWA) has gained traction and widely used for benign thyroid nodules in recent years. It offers relatively rapid and controllable heating, which may improve ablation of both cystic and solid components. However, it is more technically demanding and costlier than LIA (8, 21). Comparative data directly addressing efficacy of LIA versus MWA remain limited, particularly for nodules with an intermediate cystic proportion.

Recent research on the factors affecting physical ablation and chemical ablation efficacy identifies nodule original volume and the vascular grading as important factors influencing of PCTNs, but insufficient data on cystic proportion’s correlation with ablation efficacy remains limited. Especially for PCTNs with 50-75% proportion of cystic components, although they are mainly composed of cystic components, the solid parts cannot be ignored. Other studies have demonstrated that clinical outcomes following treatment are superior in purely cystic nodules compared to predominantly cystic ones, suggesting that the proportion of the cystic component is an important determinant of efficacy for both MWA and LIA (17, 22, 23). In fact, the proportion of cystic component of the nodule directly determines the distribution efficiency of therapeutic energy and drug penetration ability, which may have an impact on the therapeutic efficacy.

Therefore, we performed a two-center retrospective study comparing MWA and LIA for 50–75% cystic thyroid nodules and explored clinical and sonographic predictors of response, aiming to guide individualized management strategies for PCTNs in this category.

## 2 Materials and methods

### 2.1 Participants

**Figure 1:**
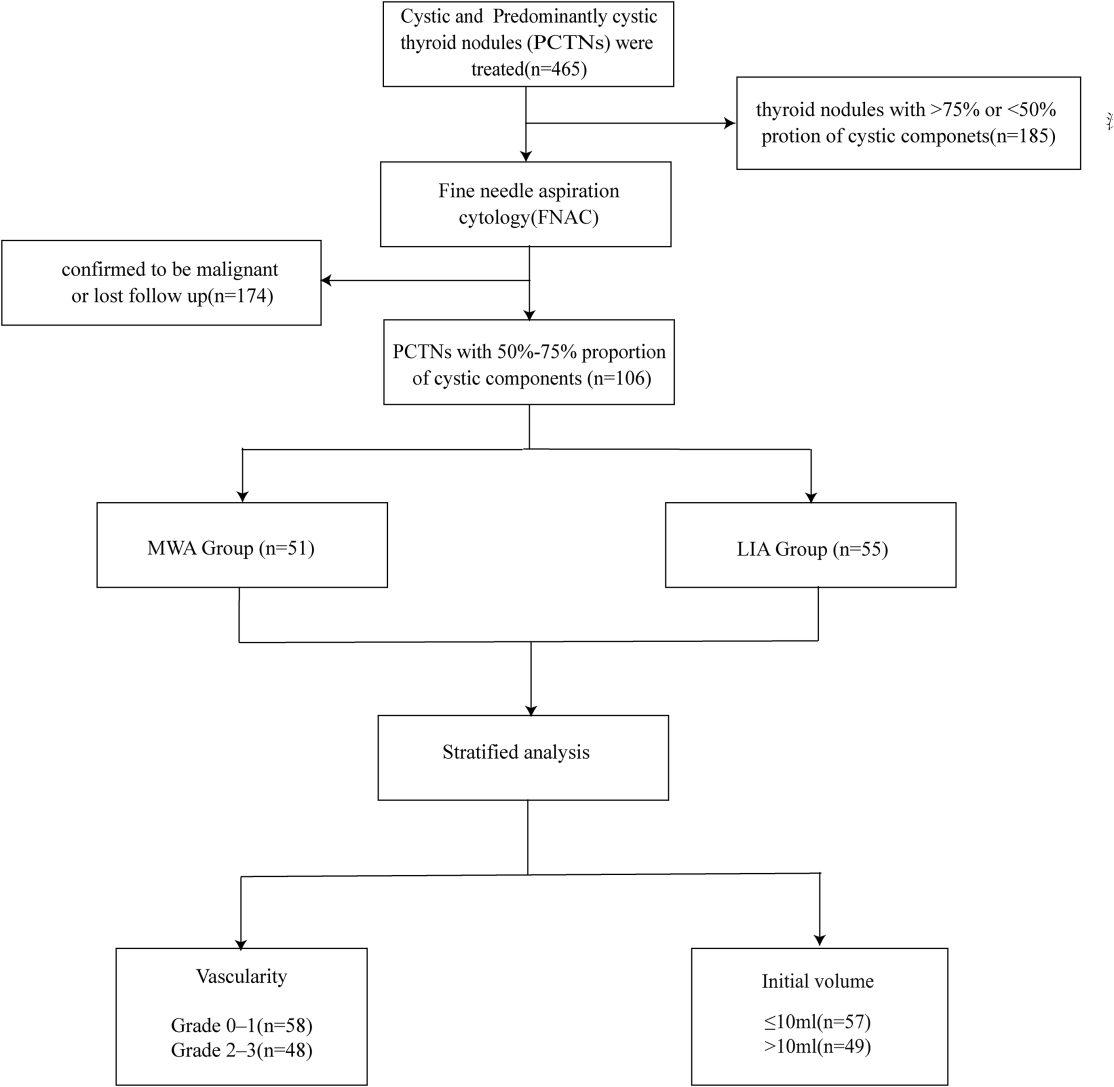
Flowchart of patient enrollment process.

This study was a two-center retrospective study approved by the Ethics Committee of XX Hospital (SWYXLL20190225-2), and written informed consent was obtained from the participants. The study selected all the patients with the diagnosis of thyroid nodules who underwent ultrasound-guided MWA or LIA treatment from April 2017 to November 2023. Patients were chosen according to the criteria (**Figure 1**) (1): predominantly cystic thyroid nodules (PCTNs, 50% <cystic component <90%) with 50%-75% proportion of cystic components (simple as 50-75% cystic thyroid nodules) (2); patients aged ≥18 years (3); benign cytological diagnosis confirmed by Fine-needle aspiration cytology (FNAC) (4); patients without coagulation disorders or serious underlying diseases,poorly controlled severe, and cardiopulmonary diseases (5); patients who have not undergone previous neck surgery (6); maximum diameter of nodules ≥2 cm. Samples with any of the following characteristics will be excluded (1): nodules confirmed to be malignant by FNAC (2); presence of bleeding-prone diseases or severe underlying diseases (3); allergic to local anesthetic drugs (4); incomplete follow-up protocol adherence (5); patients who had undergone a prior ablation procedure on the same lesion (thus excluded to ensure the homogeneity of the study cohort and to eliminate potential confounding factors).

### 2.2 Ultrasound examination

All patients underwent ultrasound and preoperative ultrasound-guided FNAC of thyroid nodules prior to therapeutic intervention. Written informed consent was provided by each patient before procedure. Thyroid ultrasound was done with MyLabTwice (Esaote, Italy) using LA523 and LA332 probes. Based on grayscale ultrasound images of the target thyroid nodule, the nodule ultrasound features characteristics (size, location, morphology and composition). The formula for calculating nodule volume: V = π×abc/6 (a is the maximum diameter and b and c are the other two perpendicular diameters). Volume reduction rate (VRR) was calculated at different time points: VRR= (initial volume-final volume)×100/initial volume. The volume of cystic fluid extracted intraoperatively was recorded: Cystic Proportion = (Vasp/Vpre)×100 (Vasp: Volume of cyst fluid completely aspirated under US guidance; Vpre: Pre-procedural volume).

### 2.3 Group criteria

Color Doppler imaging (CDFI) was utilized to assess nodule solid components vascularity, categorizing into four grades: Grade 0 (no vascularization); Grade 1 (peripheral vascularization only); Grade 2 (<50% nodular); and Grade 3 (≥50% nodular). Nodules in Grades 0–1 were further classified into the low-vascularization group, while those in Grades 2–3 were classified into the high-vascularization group. Patients were divided into two groups based on the initial volume of the nodule: those with a volume >10 ml were classified as larger nodules, and those with a volume ≤10 ml were classified as smaller nodules (18).

### 2.4 Preoperative preparation

The ablation intervention was performed using an ECO-100E microwave ablation system (ECO Medical Instruments, China) incorporating a 16-gauge (1.6 mm diameter) disposable coaxial antenna. The system operated at a frequency of 2450 MHz with power output maintained at 35 watts (W) throughout the procedure. A closed-loop saline circulation system protected surrounding tissues during energy delivery.

All ablation procedures were performed by physicians with >10 years of ultrasound experience. Preoperative preparation of patients (1): Complete laboratory tests (hematology, coagulation, liver and kidney function, infectious screening) (2). FNAC confirmed all nodules to be benign (3); Underwent B-mode ultrasonography to determine the anatomical localization, volume and cystic component proportions within the thyroid nodules.

### 2.5 Microwave ablation

**Figure 2:**
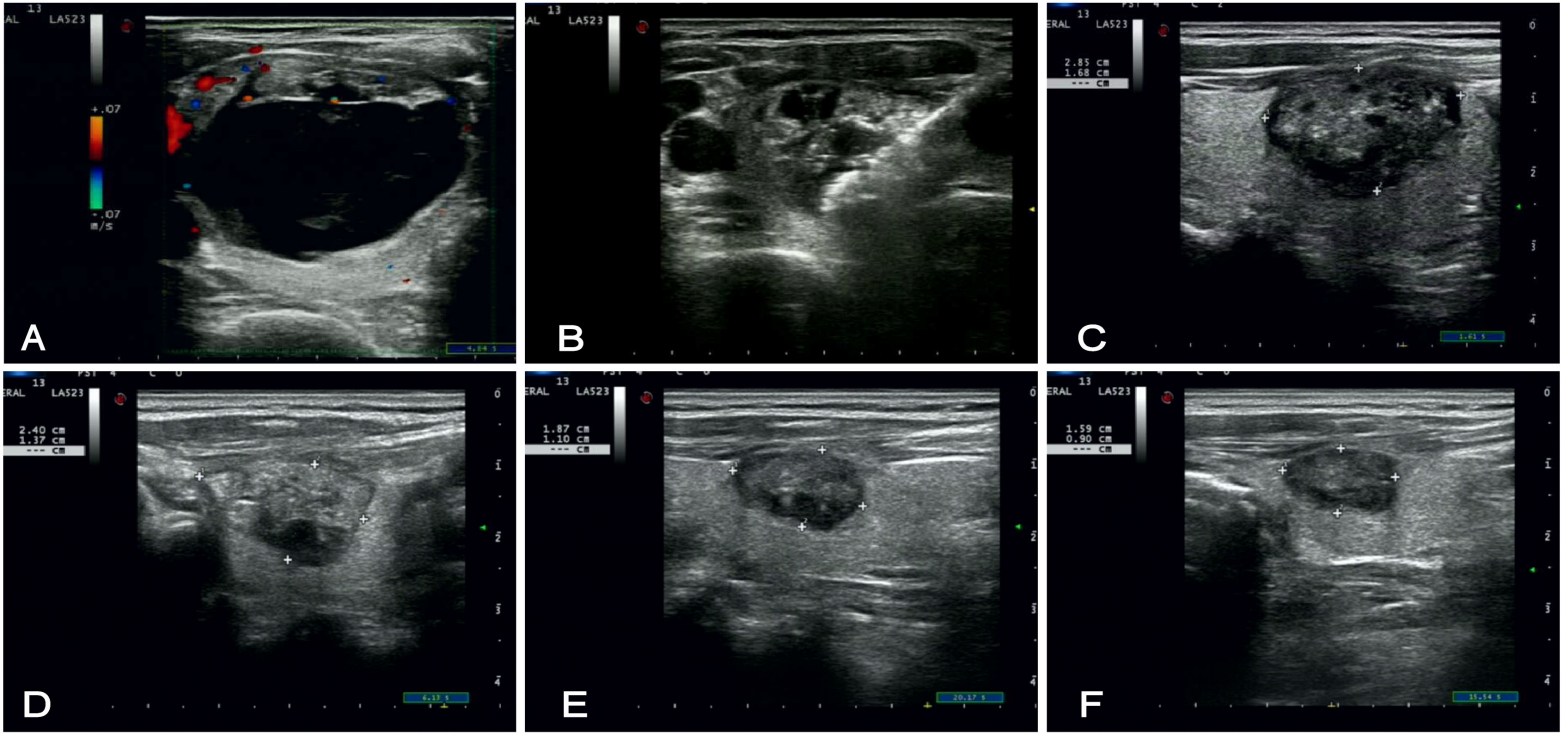
US of a 50-year-old female patient who underwent Microwave ablation for a predominantly cystic thyroid nodule in the left lobe (with a cystic component accounting for 67%), acquired at sequential time points. **(A)** Preoperative color Doppler ultrasound demonstrating a 45*26*37 mm nodule in the left lobe with punctate intraparenchymal vascularity. **(B)** Intraoperative ultrasound image depicting the target lesion during the ablation procedure. **(C–F)** Serial postoperative follow-up ultrasound images at 1, 3, 6, and 12 months, respectively, illustrating progressive nodule shrinkage with the nodule measuring 11*9 mm by the 12-month follow-up.

The patient is placed in the supine position; the neck is fully exposed and routinely sterilized. The surgeon locates the puncture site and administers local infiltration anesthesia with 2% lidocaine around the puncture site. Under ultrasound guidance, an 18-gauge coaxial needle is inserted into the cystic center of the thyroid nodule to complete aspiration. Subsequently, according to the localization of the lesion, precise hydrodissection is performed by administering a 1:1 ratio mixture of 2% lidocaine and 0.9% saline, which was injected into the anterior thyroid capsule and the muscular space to form an isolation band to protect the blood vessels, nerves, and muscular from intraoperative thermal injury Utilizing dynamic antenna technology with a power output of 35-40W, sequential ablations are performed in a layered fashion, advancing from the inferior to superior and transitioning from deep to superficial layers until the entire nodule is completely ablated (24). Contrast-enhanced ultrasound (CEUS) confirmed that the technology was successful of the procedure, including verification of adequate safety margins surrounding the treated lesion (**Figure 2**).

### 2.6 Lauromacrogol injection for ablation

**Figure 3:**
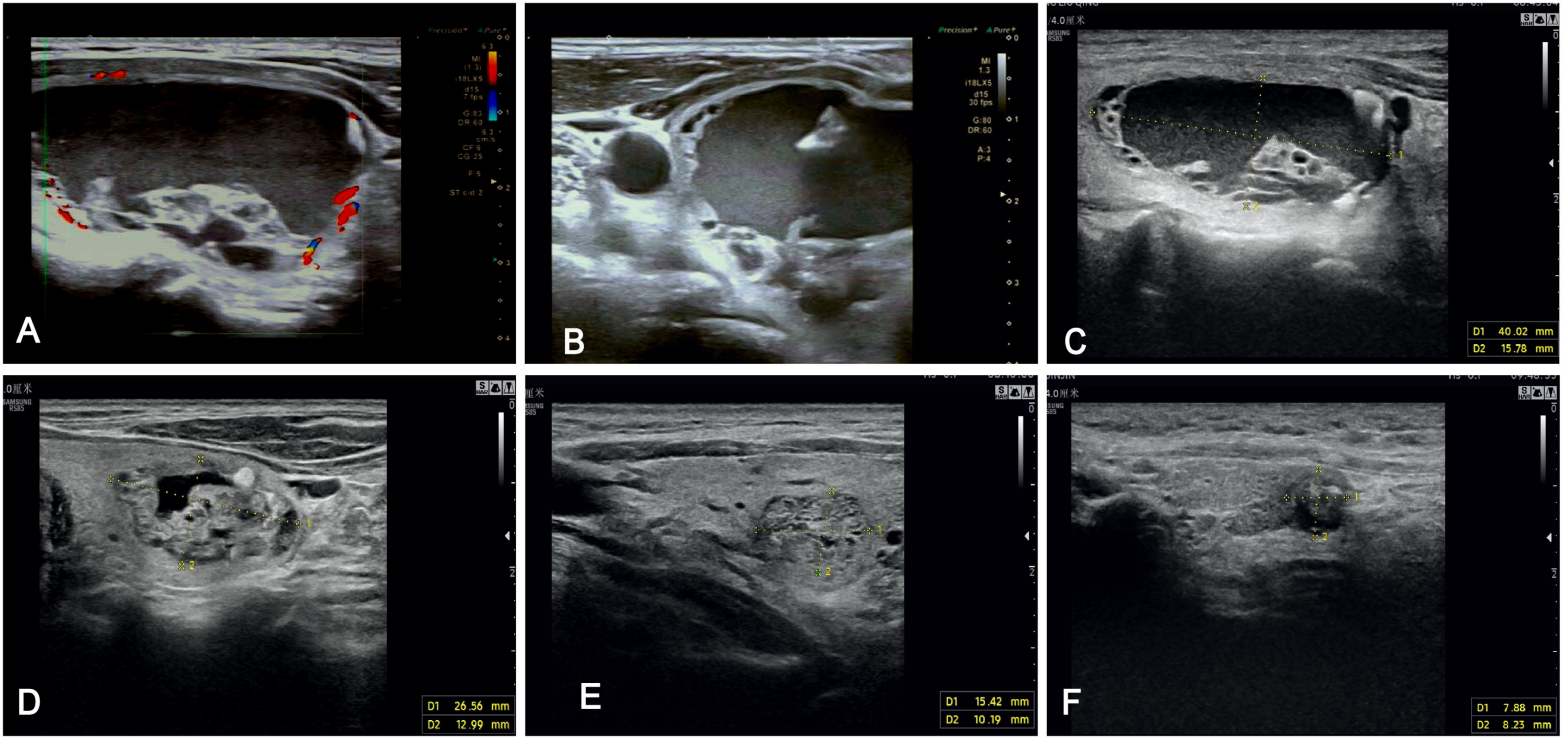
US images of a 61-year-old male patient who underwent lauromacrogol injection ablation for a predominantly cystic thyroid nodule in the left lobe (with a cystic component accounting for 71%), acquired at sequential time points. **(A)** Preoperative color Doppler ultrasound demonstrating a 35*26*43 mm predominantly cystic thyroid nodule in the left lobe with minimal perithyroidal vascularity. **(B)** Intraoperative ultrasound image depicting the target lesion during the ablation procedure. **(C–F)** Serial postoperative follow-up ultrasound images at 1, 3, 6, and 12 months, respectively, illustrating progressive nodule shrinkage with the nodule measuring 7.9*6.2 mm by the 12-month follow-up.

Standard preoperative antisepsis was performed, followed by administration of local infiltration anesthesia using 1-2% lidocaine solution. An 18-gauge needle was routinely selected, with complete aspiration of cystic fluid prior to subsequent procedures. The intracapsular fluid was completely withdrawn, followed by repeated irrigation with 0.9% sodium chloride injection or a small amount of lauromacrogol. Finally, lauromacrogol was injected into the nodule, with the injected amount generally being about 30-50% of the aspirated fluid (17, 24, 25). The volume of aspirated fluid and the administered dose of lauromacrogol were documented for each nodule (**Figure 3**).

The patient’s condition was recorded in detail intraoperatively for any intraoperative complications (intraoperative hemorrhage, peripheral organ damage, etc.), and the patient was observed in the ward after the operation for any postoperative complications.

### 2.7 Follow-up

Patients were followed up with ultrasound at regular intervals at 1, 3, and 12months postoperatively and every six months thereafter. The size of the lesion, VRR, internal structure, blood flow distribution, and complications were observed. Mild complications are referred to reactions with mild symptoms that resolve spontaneously within a short period (e.g., transient low-grade fever, moderate pain). Severe complications denote serious adverse events that may impair the function of important organs or require long-term treatment (e.g., tracheal or esophageal injury, severe infection, persistent voice abnormalities, etc.).According to the 2020 European Thyroid Association Clinical Practice Guidelines, technique efficacy is defined as VRR ≥50% of the initial nodule volume; nodule regrowth is defined as VRR ≥50% compared to the minimum recorded volume or an absolute increase in volume of ≥2 ml at a given fllow-up time point (24).

### 2.8 Statistical analysis

Statistical analysis was performed using SPSS (IBM SPSS 27.0). Continuous data were expressed as mean ± standard deviation (χ̄ ± SD). For data meeting normality assumptions, one-way analysis of covariance (ANCOVA) was employed to compare changes in nodule volume and VRR between the MWA and LIA groups, with baseline volume as a covariate. When normality was not assumed, the independent samples t-test (parametric) or Mann-Whitney U test (non-parametric) was applied based on data distribution. Categorical variables were compared using the chi-square test (χ² test). Kaplan-Meier (KM) curves were used to analyze the regrowth-free survival of patients in the two groups, and the difference in regrowth-free survival between the two groups was compared by the Log-rank test using the regrowth event as the endpoint. All P values were two-sided, and statistical significance was set at P<0.05.

## 3 Results

### 3.1 Baseline characteristics

The study enrolled 106 patients with 50-75% cystic thyroid nodules treated with MWA (51 cases) or LIA (55 cases). The baseline demographic data showed no statistical difference between the two groups (**Table 1**). There were 14 males and 37 females in the MWA group; the age range was 21–82 years, with a mean age of 53.4 ± 12.8 years. LIA group were 16 males and 39 females; the age range was 23–81 years, with a mean age of 50.2 ± 16.1 years. The mean initial nodule volume was 12.8 ± 7.3 ml in the MWA group compared to 13.6 ± 7.8 ml in the LIA group (P = 0.605). The mean follow-up time was 14.0 ± 5.2 months in the MWA group and 13.3 ± 4.0 months in the LIA group (P = 0.409), and the total follow-up time ranged from 9–25 months.

**Table 1:** Baseline characteristics of patients in MWA group and LIA group.

Characteristics	MWA group	LIA group	*P*-value
**Patient**	51	55	
**Age**	53.4 ± 12.8	50.2 ± 16.1	0.266
**Sex**			0.512
Females	37	39	
Males	14	16	
Nodules			
Maximum diameter	30.4 ± 8.1	31.3 ± 7.9	0.363
Volume	12.8 ± 7.3	13.6 ± 7.8	0.605
**Vascularity**			0.559
Grade 0–1	26/58	32/58	
Grade 2–3	25/48	23/48	
**Location**			0.848
Left	24	27	
Right	27	28	
Isthmus	1	0	
Thyroid function			
TSH	1.76	2.03	0.063
Tg-Ab	17.3	18.2	0.452
TPO-Ab	9.08	9.10	0.982
**Follow-up time** (months)	14.0 ± 5.2	13.3 ± 4.0	0.409

Values are presented as the mean ± SD.

TSH, thyroid-stimulating hormone; Tg-Ab, thyroglobulin antibodies; TPO-Ab, anti-thyroid peroxidase antibody.Bold text indicates the primary section headings for categorizing the baseline characteristics data.

### 3.2 Comparison of volume and VRR at different follow-up time points after MWA and LIA

In this study, we used ANCOVA with initial nodule volume and vascularization as a covariate to investigate the differences in nodule volume and VRR between the MWA group and the LIA group at different postoperative follow-up time points and to exclude the effect of initial nodule volume. The results showed that the nodule volume gradually decreased during the 12-month follow-up period (**Table 2**). Postoperative follow-up at 12 months showed a significant reduction in volume, the MWA group had nodules measuring 1.0 ± 1.1 ml, while the LIA group had nodules measuring 2.8 ± 4.0 ml. The corresponding follow-up time point VRR data shows that at 1-month, the MWA group had a VRR was 54.5 ± 16.0%, and the LIA group’s VRR was 55.4 ± 20.3% (F = 0.51, P = 0.950); at 3-month, the MWA group had a VRR of 75.5 ± 12.5%, while the LIA group had a VRR of 70.1 ± 17.9% (F = 4.52, P = 0.360); at 6-month, the MWA group had a VRR of 86.0 ± 9.2%, and the LIA group had a VRR of 78.0 ± 19.4% (F = 8.64, P = 0.004); at 12 months post-operation, the MWA group had a VRR was 91.5 ± 9.8%, and the LIA group’s VRR was 81.1 ± 2.4% (F = 9.83, P = 0.002).

**Table 2:** Volume and volume reduction rate (VRR) comparison of MWA and LIA groups at different follow-up times.

Follow-up	Group	Volume (mean ± SD)	VRR (%) (mean ± SD)	(95%CI)	F	*P*-value
1 month	MWA	5.4 ± 2.8	54.5 ± 16.0	(0.498,0.598)	0.51	0.950
	LIA	6.3 ± 5.2	55.4 ± 20.3	(0.507,0.604)		
3 months	MWA	2.8 ± 1.8	75.5 ± 12.5	(0.715,0.977)	4.52	0.360
	LIA	4.4 ± 4.3	70.1 ± 17.9	(0.662,0,743)		
6 months	MWA	1.6 ± 1.2	86.0 ± 9.2	(0.819,0.903)	8.64	0.004*
	LIA	3.3 ± 3.9	78.0 ± 19.4	(0.743,0.823)		
12 months	MWA	1.0 ± 1.1	91.5 ± 9.8	(0.864,0.968)	9.83	0.002*
	LIA	2.8 ± 4.0	81.1 ± 2.4	(0.763,0.862)		

Values are presented as the mean ± SD. Analysis of Variance, ANOVA. MWA, microwave ablation; LIA, lauromacrogol injection for ablation; CI, confidence interval.Bold font with asterisk (*): P < 0.05 (statistically significant).

Furthermore, ANCOVA showed that the main effect test for initial nodule volume was not statistically significant (P>0.05), but the interaction test with treatment method indicated a significant interaction (P<0.05 at time points VRR1/3/6/12, etc.); the main effect test for vascularization degree at VRR1/3/6/12 was statistically significant (P<0.05), but the interaction with treatment method was not statistically significant (P<0.05) (**Supplementary Table 1**).

### 3.3 Comparison with treatment effectiveness (VRR>50%) at 6-month and 12-month follow-up between the MWA group and LIA group

This study defines VRR>50% as effective ablation and evaluates the efficacy of the MWA group and LIA group at 6 and 12 months postoperatively (**Table 3**). The 6-month postoperative follow-up showed that 50 patients (98.0%) in the MWA group achieved a VRR>50% and 1 patient (1.9%) had a VRR<50%; 47 patients (85.4%) in the LIA group achieved a VRR>50%, and 8 (14.5%) achieved a VRR<50% (P = 0.003). At 12 months postoperative follow-up, 49 patients (96.1%) of the MWA group achieved VRR>50% and 2 (3.9%) did not achieved VRR<50%, while 46 patients (81.8%) in the LIA group achieved VRR>50% and 10 (18.2%) did not achieved VRR<50% (P = 0.018). In conclusion, MWA group had significantly higher proportion of patients with VRR>50% (effective ablation) than LIA group at the 6-month and 12-month postoperative follow-up.

**Table 3:** Comparison with treatment effectiveness (VRR>50%) between the MWA group and LIA group.

Follow-up	6 months		12 months	
Groups	VRR>50%	VRR<50%	VRR>50%	VRR<50%
MWA	50(98.0%)	1(1.9%)	49(96.1%)	2(3.9%)
LIA	47(85.4%)	8(14.5%)	46(81.8%)	10(18.2%)
*P*-Value		0.003*		0.018*

Values are presented as the mean ± SD. MWA, microwave ablation; LIA, lauromacrogol injection for ablation.Bold font with asterisk (*): P < 0.05 (statistically significant).

### 3.4 Stratified analysis by nodule size and vascularization

This study further performed a stratified analysis based on the degree of vascularization and initial nodule volume to explore the heterogeneity of the two groups’ impact on the volume reduction rate (VRR) (**Table 4**). Within the stratified analysis of vascularization, there was no statistically significant difference between the two groups at 1 and 3 months (P<0.05); at 6 and 12 months, the VRR of the microwave ablation (MWA) group was significantly higher than that of the lauromacrogol injection for ablation (LIA) group (P<0.05).When stratified by initial volume, in the ≤10 ml group, there were no significant differences in VRR between MWA and LIA at 1, 3, 6, and 12 months (P>0.05); in the >10 ml group, MWA showed significantly better VRR than LIA at 3, 6, and 12 months (P<0.05).

**Table 4:** Subgroup analysis results by nodule size and vascularization.

Subgroup	Groups	VRR% (1-month)	*P*-value	VRR% (3-month)	*P*-value	VRR% (6-month)	*P*-Value	VRR% (12-month)	*P*-value
Vascularity									
Grade 0–1	MWA	57.6 ± 15.6	0.987	78.1 ± 11.3	0.125	88.3 ± 7.6	0.042*	94.6 ± 4.5	0.019*
	LIA	57.7 ± 21.1		72.4 ± 15.8		80.7 ± 18.5		85.2 ± 20.8	
Grade 2–3	MWA	51.6 ± 16.1	0.899	72.9 ± 13.3	0.196	83.6 ± 10.3	0.044*	88.5 ± 12.4	0.030*
	LIA	52.2 ± 19.4		66.5 ± 20.5		74.2 ± 20.5		75.3 ± 27.3	
Initial volume									
≤10ml	MWA	51.1 ± 15.3	0.254	71.8 ± 11.8	0.467	83.2 ± 9.6	0.302	90.3 ± 11.2	0.122
	LIA	54.5 ± 20.5		71.5 ± 18.1		81.0 ± 19,2		83.7 ± 25.3	
>10ml	MWA	57.8 ± 16.3	0.719	78.9 ± 12.4	0.016*	88.5 ± 8.4	0.004*	92.7 ± 8.4	0.002*
	LIA	56.0 ± 20.6		68.9 ± 18.1		75.7 ± 19,7		79.0 ± 19.7	

Values are presented as the mean ± SD. MWA, microwave ablation; LIA, lauromacrogol injection for ablation.Bold font with asterisk (*): P < 0.05 (statistically significant).

### 3.5 Comparison of nodule recurrence and complications between the MWA and LIA groups

**Figure 4:**
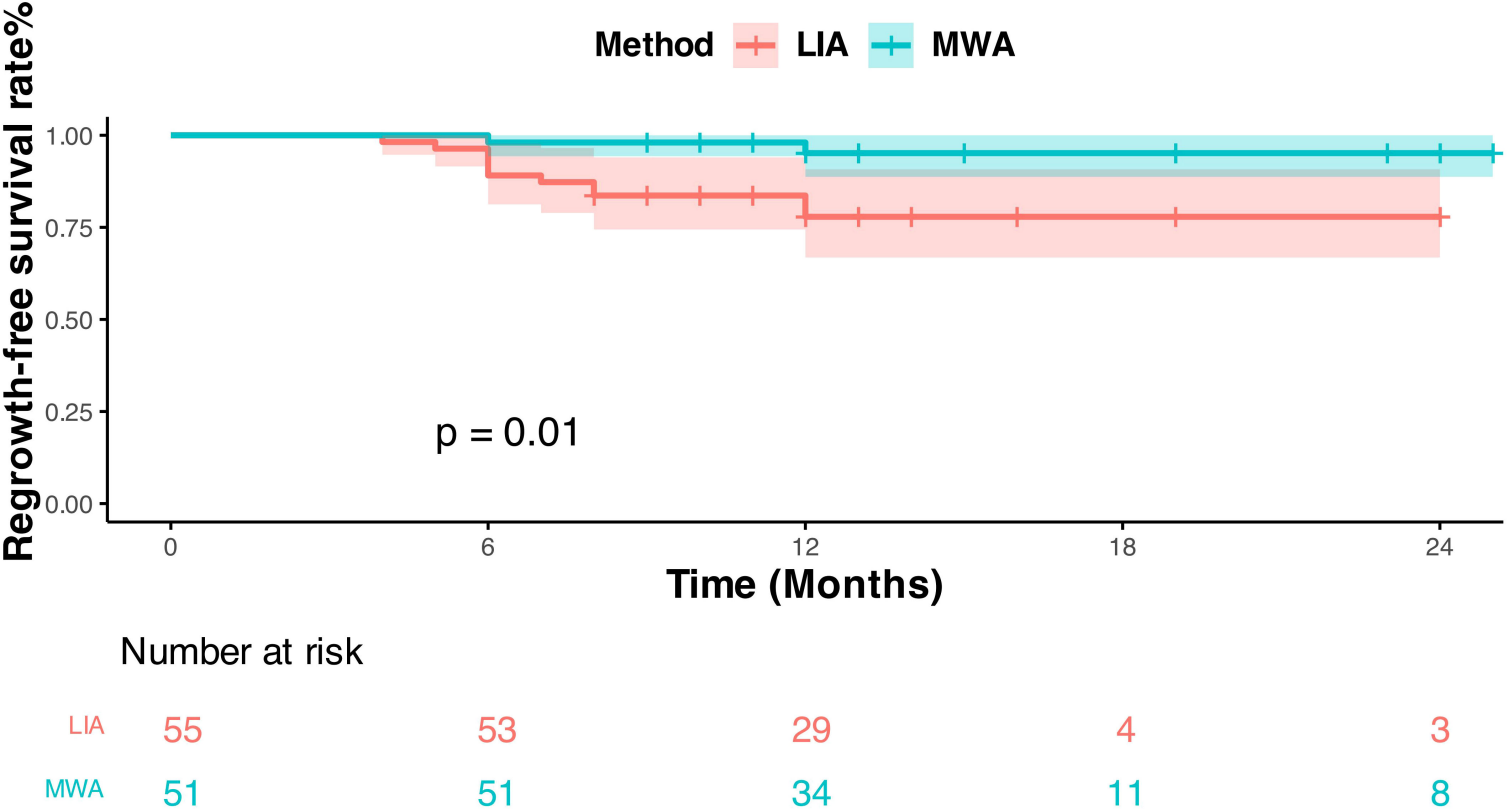
This figure uses Kaplan-Meier curves to compare post-treatment regrowth-free survival (RFS) rates between LIA and MWA. The Y-axis shows RFS rate (%), and the X-axis shows follow-up time (months); the red curve represents the LIA group, and the cyan curve represents the MWA group. Results show the MWA group has a significantly higher RFS rate than the LIA group (log-rank p = 0.01). Below the figure, The “Number at risk” displays the number of patients still under observation in both groups at different follow-up time points.

The study analyzed the postoperative occurrence of 50-75% cystic thyroid nodules regeneration and complications at different time points between the MWA group and the LIA group (**Table 5**). The results of nodule regeneration analysis showed that at <6 months, there were no cases of nodule regeneration in the MWA group (0/51, 0%), and two cases (2/55, 3.6%) appeared in the LIA group; at 6 -month, there was one case (1/51, 1.9%) in the MWA group and seven cases (7/55, 16.3%) in the LIA group; at 12-month, there was one case (1/51, 1.9%) in the MWA group and 3 cases (3/55, 5.4%) in the LIA group. Overall, the nodule regeneration rate was 3.9% (2/51) in the MWA group and 20% (11/55) in the LIA group (P = 0.006). All complications recovered within 3 months. KM curve analysis showed (**Figure 4**) that there was a statistically significant difference in regrowth-free survival between the MWA group and the LIA group, using regrowth as the endpoint (Log-rank test P = 0.010). During the follow-up period, the overall regrowth-free survival rate was significantly higher in the MWA group than in the LIA group.

**Table 5:** Regrowth rates and complications comparison between MWA and LIA groups.

Follow-up outcomes	MWA	LIA	Total	*P-*value
Regrowth				
<6 months	0/51(0)	2/55(3.6%)	2	
6 months	1/51(1.9%)	7/55(16.3%)	8	
12 months	1/51(1.9%)	3/55(5.4%)	4	
Total	2/51(3.9%)	11/55(20%)	13	0.006*
Complication				
Major complication	0	0		
Minor complication	4	3		
Total	4/51(78.4%)	3/55(54.5%)	7	0.709

Values are presented as the mean ± SD. MWA, microwave ablation; LIA, lauromacrogol injection for ablation.Bold font with asterisk (*): P < 0.05 (statistically significant).

Regarding complications, no serious complications occurred in both groups. Among them, four minor complications occurred in the MWA group (4/51) and three in the LIA group (3/55), and the difference in the overall complications rate between the two groups was not statistically significant (P = 0.709).

## 4 Discussion

In our research, the results showed that both Microwave Ablation (MWA) and lauromacrogol injection for ablation (LIA) exhibit efficacy and safety in the treatment of 50-75% cystic thyroid nodules. Recent studies have also confirmed that MWA is safe and effective for the treatment of benign thyroid nodules. In the study by Luo et al, 19 patients with PCTNs demonstrated VRR of 83.9% at 6 months and 88.9% at 12 months postoperatively, which is like our outcomes. The findings show that microwave ablation is safe and effective for the management of PCTNs (26). LIA has attracted increasing attention in the treatment of cystic thyroid nodules in recent years due to its well-documented efficacy and minimal adverse effects (15, 27). In Dong et al.’s study, they considered VRR >50% from 6 to 12 months as effective treatment. Their findings show that the ablation of cystic nodules was better than that of cystic primary nodules (therpeutic success rate: 91.6% vs. 75.9%) (17). Our study demonstrated outcomes consistent with previous research. Among the cohort, 55 nodules had a VRR >50% at 6 or 12 months postoperatively. The VRR reached 78.0 ± 19.4% and 81.1 ± 2.4% at months 6 and 12, respectively, with a treatment success rate of 81.8% (46/55), which was higher than that reported in Dong’s study. This may be attributed to study inclusion of nodules with only single ablation within the 12-months postoperative.

Our results show that with longer follow-up, VRR gradually increased in both MWA and LIA groups. At 3 months postoperatively, VRR was 75.5 ± 12.5% (MWA) vs 70.1 ± 17.9% (LIA), with no significant difference (P>0.05). However, at 6 months, MWA reached 86.0% ± 9.2%, significantly higher than LIA’s 78.0 ± 19.4% (P<0.05); at 12 months, MWA further increased to 91.5 ± 9.8%, vs LIA’s 81.1 ± 2.4% (P<0.05).This result suggests that for50-75% cystic thyroid nodules, MWA showed greater efficacy in reducing nodule volume over the medium and long term, as its thermal effect more extensively and uniformly. In contrast, LIA exhibits relatively limited transmission and range of action which may cause incomplete ablation and thereby hinder nodule volume reduction (19, 23, 28).

This study provides guidance for the selection of treatment for 50-75% cystic thyroid nodules. through stratified analysis of vascularization and initial nodule volume. In both Grade 0–1 and Grade 2–3 subgroups, MWA showed significantly higher VRR than LIA in 6 and 12 months (P<0.05). This suggests that the long-term efficacy advantage of MWA gradually becomes evident across nodules of different vascularization grades. It is noteworthy that the VRR values of MWA in the 2–3 grade subgroup were lower than those in the 0–1 grade subgroup. Combined with the significant results of ANCOVA testing the between-group effects of vascularization levels, it suggests that while vascularization levels significantly impact efficacy, they do not significantly modulate the differences in efficacy among different treatment modalities. This indicates that for patients requiring long-term control of nodules in a clinical setting, MWA may be a better option regardless of the degree of nodule vascularization. It is worth noting that in the 2–3 grade subgroup, the VRR is generally higher than in the 0–1 grade subgroup, indicating that the degree of vascularization may be a potential factor affecting the efficacy of ablation. The stratified analysis results of the initial nodule volume size showed that there was no significant difference in VRR at all time points between MWA and LIA in the ≤10ml subgroup (P>0.05). However, overall, the VRR in the MWA group was still superior to that in the LIA group. In the >10 ml subgroup, the VRR of MWA was significantly superior to that of LIA at 3 months, 6 months, and 12 months (P<0.05). Larger nodules have more complex blood supply and structural features, and LIA is limited by the penetration range of chemical agents, so MWA thermal ablation is more suitable for dealing with larger nodules. Therefore, for patients with 50-75% cystic thyroid nodules >10 ml, MWA therapy should be prioritized; for nodules ≤10 ml, decisions should be made based on a comprehensive assessment of patient tolerance, economic costs, and other factors.

The results of the analysis of nodule regeneration and the occurrence of complications in patients in the MWA group and the LIA group showed that nodule regrowth mostly occurred in the 6th month after ablation, suggesting that the 6th month after surgery may be a critical point for nodule regrowth, and that the monitoring of patients should be strengthened. During the follow-up period, the nodule regrowth rate was 3.9% (2/51) in the MWA group and 20% (11/55) in the LIA group, demonstrating a statistically significant difference between the two groups (P = 0.006). The overall regrowth-free survival rate was notably higher in the MWA group compared to the LIA group, suggesting that MWA may be more effective than LIA in reducing the risk of regrowth for the treatment of 50-75% cystic thyroid nodules. This may be related to the mechanism of action of MWA.MWA caused irreversible coagulative necrosis of nodule tissues, whereas LIA causes ischemia and fibrosis of the nodules mainly by destroying vascular endothelial cells, which may result in residual nodule tissue and increase the risk of regeneration (8, 19, 28). Notably, previous studies have indicated that MWA entails a higher cost (29). In this study, MWA demonstrated excellent therapeutic efficacy and a recurrence rate significantly lower than that of LIA. The reduced recurrence rate can reduce the need for repeat surgeries, the number of additional surgeries, and the duration of long-term follow-up, as well as alleviate patients’ anxiety about the disease in the, thereby improving the overall benefits in the long run. The initial cost and long-term benefits have clinical relevance. Combined with the results of our stratified analysis, it is helpful for clinicians to select better treatment strategies.For instance, MWA can be prioritized for the treatment of such larger nodules with higher vascularization. Owing to the limitations of this study, no further research has been conducted on overall benefits. In the future, a long-term prospective study can be conducted to further explore its results in terms of overall benefits.Regarding complications, both groups had no major complications, with no statistically significant difference in total complication rates (P = 0.709), indicating comparable safety and good tolerability consistent with other studies.

This study has several limitations. First, the study design is retrospective analysis, which may have selection bias and information bias, affecting the reliability of the results. Second, the sample size is relatively small, which may lead to insufficient representativeness of the research findings.

In conclusion, the results showed that for 50-75% cystic thyroid nodules, MWA demonstrated more significant volume reduction and better therapeutic efficacy during follow-up, with a significantly lower regrowth rate compared to the LIA group. Notably, MWA achieved better outcomes than LIA when treating larger nodules. In terms of complications, the MWA group had a slightly higher number of complications than the LIA group, though without significant statistical difference, possibly due to the small sample size. However, due to limitations including retrospective design, sample size, and follow-up duration, these conclusions require further validation through larger-scale, prospective, multi-center randomized controlled trials.

## Data Availability

The datasets presented in this article are not readily available because the clinical data used in this study on thyroid cystic-solid nodule ablation are restricted to protect patient confidentiality, as mandated by the ethical approval (Approval No.: [SWYXLL20190225–2]) obtained from the Affiliated Hospital of Jiangsu University. Public release of the dataset is prohibited due to the inclusion of identifiable patient information (e.g., thyroid function test results, ultrasound reports, and ablation outcome data). Anonymized data for result verification or secondary analysis are available from the corresponding author upon reasonable request, subject to approval by the institutional ethical review committee of the Affiliated Hospital of Jiangsu University. Researchers must provide a detailed research proposal and agree to strict data security and confidentiality requirements. Requests to access the datasets should be directed to Jiayan Bao, baojiayan1004@163.com.
