# Supplementary material for: Comparison of microwave ablation versus lauromacrogol injection ablation for 50–75% cystic thyroid nodules: a two-center retrospective study
Source: Front Endocrinol (Lausanne). 2026 Mar 10;17:1751988. doi: 10.3389/fendo.2026.1751988 (PMC13008710; doi:10.3389/fendo.2026.1751988)
Supplement: Supplementary file 1 [file Table1.docx]

| **Supplementary Table 1.Test of Between-Subjects Effects and Test of Interaction Terms** | | | | | |
| --- | --- | --- | --- | --- | --- |
|  | **Type III sum of squares** | **df** | **MS** | **F** | ***P*-Value** |
| **Vascularity** | .131 | 1 | .131 | 4.089 | .046 |
|  | .127 | 1 | .127 | 5.855 | .017 |
|  | .123 | 1 | .123 | 5.673 | .019 |
|  | .203 | 1 | .203 | 6.152 | .015 |
| **V0** | .009 | 1 | .009 | .289 | .592 |
|  | .000 | 1 | .000 | .009 | .925 |
|  | .008 | 1 | .008 | .363 | .548 |
|  | .013 | 1 | .013 | .396 | .530 |
| **group * V0** | .241 | 1 | .241 | 7.534 | .007 |
|  | .278 | 1 | .278 | 12.796 | <.001 |
|  | .208 | 1 | .208 | 9.563 | .003 |
|  | .094 | 1 | .094 | 2.850 | .045 |
| **group * Vascularity** | .001 | 1 | .001 | .033 | .856 |
|  | .000 | 1 | .000 | .012 | .912 |
|  | .003 | 1 | .003 | .144 | .705 |
|  | .013 | 1 | .013 | .381 | .538 |

MS,mean square; df, degree of freedom
